# A survey of best practices for RNA-seq data analysis

**DOI:** 10.1186/s13059-016-0881-8

**Published:** 2016-01-26

**Authors:** Ana Conesa, Pedro Madrigal, Sonia Tarazona, David Gomez-Cabrero, Alejandra Cervera, Andrew McPherson, Michał Wojciech Szcześniak, Daniel J. Gaffney, Laura L. Elo, Xuegong Zhang, Ali Mortazavi

**Affiliations:** Institute for Food and Agricultural Sciences, Department of Microbiology and Cell Science, University of Florida, Gainesville, FL 32603 USA; Centro de Investigación Príncipe Felipe, Genomics of Gene Expression Laboratory, 46012 Valencia, Spain; Wellcome Trust Sanger Institute, Wellcome Trust Genome Campus, Hinxton, Cambridge, CB10 1SA UK; Wellcome Trust-Medical Research Council Cambridge Stem Cell Institute, Anne McLaren Laboratory for Regenerative Medicine, Department of Surgery, University of Cambridge, Cambridge, CB2 0SZ UK; Department of Applied Statistics, Operations Research and Quality, Universidad Politécnica de Valencia, 46020 Valencia, Spain; Unit of Computational Medicine, Department of Medicine, Karolinska Institutet, Karolinska University Hospital, 171 77 Stockholm, Sweden; Center for Molecular Medicine, Karolinska Institutet, 17177 Stockholm, Sweden; Unit of Clinical Epidemiology, Department of Medicine, Karolinska University Hospital, L8, 17176 Stockholm, Sweden; Science for Life Laboratory, 17121 Solna, Sweden; Systems Biology Laboratory, Institute of Biomedicine and Genome-Scale Biology Research Program, University of Helsinki, 00014 Helsinki, Finland; School of Computing Science, Simon Fraser University, Burnaby, V5A 1S6 BC Canada; Department of Bioinformatics, Institute of Molecular Biology and Biotechnology, Adam Mickiewicz University in Poznań, 61-614 Poznań, Poland; Turku Centre for Biotechnology, University of Turku and Åbo Akademi University, FI-20520 Turku, Finland; Key Lab of Bioinformatics/Bioinformatics Division, TNLIST and Department of Automation, Tsinghua University, Beijing, 100084 China; School of Life Sciences, Tsinghua University, Beijing, 100084 China; Department of Developmental and Cell Biology, University of California, Irvine, Irvine, CA 92697-2300 USA; Center for Complex Biological Systems, University of California, Irvine, Irvine, CA 92697 USA

## Abstract

RNA-sequencing (RNA-seq) has a wide variety of applications, but no single analysis pipeline can be used in all cases. We review all of the major steps in RNA-seq data analysis, including experimental design, quality control, read alignment, quantification of gene and transcript levels, visualization, differential gene expression, alternative splicing, functional analysis, gene fusion detection and eQTL mapping. We highlight the challenges associated with each step. We discuss the analysis of small RNAs and the integration of RNA-seq with other functional genomics techniques. Finally, we discuss the outlook for novel technologies that are changing the state of the art in transcriptomics.

**Electronic supplementary material:**

The online version of this article (doi:10.1186/s13059-016-0881-8) contains supplementary material, which is available to authorized users.

## Background

Transcript identification and the quantification of gene expression have been distinct core activities in molecular biology ever since the discovery of RNA’s role as the key intermediate between the genome and the proteome. The power of sequencing RNA lies in the fact that the twin aspects of discovery and quantification can be combined in a single high-throughput sequencing assay called RNA-sequencing (RNA-seq). The pervasive adoption of RNA-seq has spread well beyond the genomics community and has become a standard part of the toolkit used by the life sciences research community. Many variations of RNA-seq protocols and analyses have been published, making it challenging for new users to appreciate all of the steps necessary to conduct an RNA-seq study properly.

There is no optimal pipeline for the variety of different applications and analysis scenarios in which RNA-seq can be used. Scientists plan experiments and adopt different analysis strategies depending on the organism being studied and their research goals. For example, if a genome sequence is available for the studied organism, it should be possible to identify transcripts by mapping RNA-seq reads onto the genome. By contrast, for organisms without sequenced genomes, quantification would be achieved by first assembling reads de novo into contigs and then mapping these contigs onto the transcriptome. For well-annotated genomes such as the human genome, researchers may choose to base their RNA-seq analysis on the existing annotated reference transcriptome alone, or might try to identify new transcripts and their differential regulation. Furthermore, investigators might be interested only in messenger RNA isoform expression or microRNA (miRNA) levels or allele variant identification. Both the experimental design and the analysis procedures will vary greatly in each of these cases. RNA-seq can be used solo for transcriptome profiling or in combination with other functional genomics methods to enhance the analysis of gene expression. Finally, RNA-seq can be coupled with different types of biochemical assay to analyze many other aspects of RNA biology, such as RNA–protein binding, RNA structure, or RNA–RNA interactions. These applications are, however, beyond the scope of this review as we focus on ‘typical’ RNA-seq.

**Fig. 1:**
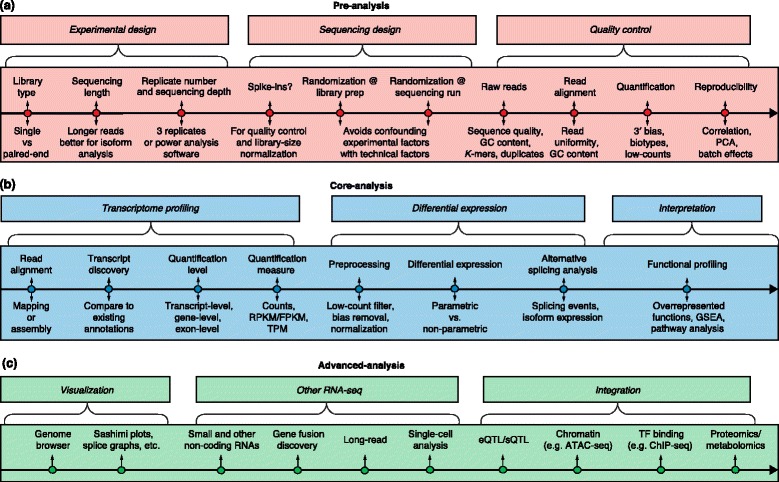
A generic roadmap for RNA-seq computational analyses. The major analysis steps are listed above the lines for pre-analysis, core analysis and advanced analysis. The key analysis issues for each step that are listed below the lines are discussed in the text. **a** Preprocessing includes experimental design, sequencing design, and quality control steps. **b** Core analyses include transcriptome profiling, differential gene expression, and functional profiling. **c** Advanced analysis includes visualization, other RNA-seq technologies, and data integration. Abbreviations: *ChIP-seq* Chromatin immunoprecipitation sequencing, *eQTL* Expression quantitative loci, *FPKM* Fragments per kilobase of exon model per million mapped reads, *GSEA* Gene set enrichment analysis, *PCA* Principal component analysis, *RPKM* Reads per kilobase of exon model per million reads, *sQTL* Splicing quantitative trait loci, *TF* Transcription factor, *TPM* Transcripts per million

Every RNA-seq experimental scenario could potentially have different optimal methods for transcript quantification, normalization, and ultimately differential expression analysis. Moreover, quality control checks should be applied pertinently at different stages of the analysis to ensure both reproducibility and reliability of the results. Our focus is to outline current standards and resources for the bioinformatics analysis of RNA-seq data. We do not aim to provide an exhaustive compilation of resources or software tools nor to indicate one best analysis pipeline. Rather, we aim to provide a commented guideline for RNA-seq data analysis. Figure 1 depicts a generic roadmap for experimental design and analysis using standard Illumina sequencing. We also briefly list several data integration paradigms that have been proposed and comment on their potential and limitations. We finally discuss the opportunities as well as challenges provided by single-cell RNA-seq and long-read technologies when compared to traditional short-read RNA-seq.

## Experimental design

A crucial prerequisite for a successful RNA-seq study is that the data generated have the potential to answer the biological questions of interest. This is achieved by first defining a good experimental design, that is, by choosing the library type, sequencing depth and number of replicates appropriate for the biological system under study, and second by planning an adequate execution of the sequencing experiment itself, ensuring that data acquisition does not become contaminated with unnecessary biases. In this section, we discuss both considerations.

One important aspect of the experimental design is the RNA-extraction protocol used to remove the highly abundant ribosomal RNA (rRNA), which typically constitutes over 90 % of total RNA in the cell, leaving the 1–2 % comprising messenger RNA (mRNA) that we are normally interested in. For eukaryotes, this involves choosing whether to enrich for mRNA using poly(A) selection or to deplete rRNA. Poly(A) selection typically requires a relatively high proportion of mRNA with minimal degradation as measured by RNA integrity number (RIN), which normally yields a higher overall fraction of reads falling onto known exons. Many biologically relevant samples (such as tissue biopsies) cannot, however, be obtained in great enough quantity or good enough mRNA integrity to produce good poly(A) RNA-seq libraries and therefore require ribosomal depletion. For bacterial samples, in which mRNA is not polyadenylated, the only viable alternative is ribosomal depletion. Another consideration is whether to generate strand-preserving libraries. The first generation of Illumina-based RNA-seq used random hexamer priming to reverse-transcribe poly(A)-selected mRNA. This methodology did not retain information contained on the DNA strand that is actually expressed [1] and therefore complicates the analysis and quantification of antisense or overlapping transcripts. Several strand-specific protocols [2], such as the widely used dUTP method, extend the original protocol by incorporating UTP nucleotides during the second cDNA synthesis step, prior to adapter ligation followed by digestion of the strand containing dUTP [3]. In all cases, the size of the final fragments (usually less than 500 bp for Illumina) will be crucial for proper sequencing and subsequent analysis. Furthermore, sequencing can involve single-end (SE) or paired-end (PE) reads, although the latter is preferable for de novo transcript discovery or isoform expression analysis [4, 5]. Similarly, longer reads improve mappability and transcript identification [5, 6]. The best sequencing option depends on the analysis goals. The cheaper, short SE reads are normally sufficient for studies of gene expression levels in well-annotated organisms, whereas longer and PE reads are preferable to characterize poorly annotated transcriptomes.

Another important factor is sequencing depth or library size, which is the number of sequenced reads for a given sample. More transcripts will be detected and their quantification will be more precise as the sample is sequenced to a deeper level [1]. Nevertheless, optimal sequencing depth again depends on the aims of the experiment. While some authors will argue that as few as five million mapped reads are sufficient to quantify accurately medium to highly expressed genes in most eukaryotic transcriptomes, others will sequence up to 100 million reads to quantify precisely genes and transcripts that have low expression levels [7]. When studying single cells, which have limited sample complexity, quantification is often carried out with just one million reads but may be done reliably for highly expressed genes with as few as 50,000 reads [8]; even 20,000 reads have been used to differentiate cell types in splenic tissue [9]. Moreover, optimal library size depends on the complexity of the targeted transcriptome. Experimental results suggest that deep sequencing improves quantification and identification but might also result in the detection of transcriptional noise and off-target transcripts [10]. Saturation curves can be used to assess the improvement in transcriptome coverage to be expected at a given sequencing depth [10].

Finally, a crucial design factor is the number of replicates. The number of replicates that should be included in a RNA-seq experiment depends on both the amount of technical variability in the RNA-seq procedures and the biological variability of the system under study, as well as on the desired statistical power (that is, the capacity for detecting statistically significant differences in gene expression between experimental groups). These two aspects are part of power analysis calculations (Fig. 1a; Box 1).

The adequate planning of sequencing experiments so as to avoid technical biases is as important as good experimental design, especially when the experiment involves a large number of samples that need to be processed in several batches. In this case, including controls, randomizing sample processing and smart management of sequencing runs are crucial to obtain error-free data (Fig. 1a; Box 2).

## Analysis of the RNA-seq data

The actual analysis of RNA-seq data has as many variations as there are applications of the technology. In this section, we address all of the major analysis steps for a typical RNA-seq experiment, which involve quality control, read alignment with and without a reference genome, obtaining metrics for gene and transcript expression, and approaches for detecting differential gene expression. We also discuss analysis options for applications of RNA-seq involving alternative splicing, fusion transcripts and small RNA expression. Finally, we review useful packages for data visualization.

### Quality-control checkpoints

The acquisition of RNA-seq data consists of several steps — obtaining raw reads, read alignment and quantification. At each of these steps, specific checks should be applied to monitor the quality of the data (Fig. 1a).

#### Raw reads

Quality control for the raw reads involves the analysis of sequence quality, GC content, the presence of adaptors, overrepresented *k*-mers and duplicated reads in order to detect sequencing errors, PCR artifacts or contaminations. Acceptable duplication, *k*-mer or GC content levels are experiment- and organism-specific, but these values should be homogeneous for samples in the same experiments. We recommend that outliers with over 30 % disagreement to be discarded. FastQC [11] is a popular tool to perform these analyses on Illumina reads, whereas NGSQC [12] can be applied to any platform. As a general rule, read quality decreases towards the 3’ end of reads, and if it becomes too low, bases should be removed to improve mappability. Software tools such as the FASTX-Toolkit [13] and Trimmomatic [14] can be used to discard low-quality reads, trim adaptor sequences, and eliminate poor-quality bases.

#### Read alignment

Reads are typically mapped to either a genome or a transcriptome, as will be discussed later. An important mapping quality parameter is the percentage of mapped reads, which is a global indicator of the overall sequencing accuracy and of the presence of contaminating DNA. For example, we expect between 70 and 90 % of regular RNA-seq reads to map onto the human genome (depending on the read mapper used) [15], with a significant fraction of reads mapping to a limited number of identical regions equally well (‘multi-mapping reads’). When reads are mapped against the transcriptome, we expect slightly lower total mapping percentages because reads coming from unannotated transcripts will be lost, and significantly more multi-mapping reads because of reads falling onto exons that are shared by different transcript isoforms of the same gene.

Other important parameters are the uniformity of read coverage on exons and the mapped strand. If reads primarily accumulate at the 3’ end of transcripts in poly(A)-selected samples, this might indicate low RNA quality in the starting material. The GC content of mapped reads may reveal PCR biases. Tools for quality control in mapping include Picard [16], RSeQC [17] and Qualimap [18].

#### Quantification

Once actual transcript quantification values have been calculated, they should be checked for GC content and gene length biases so that correcting normalization methods can be applied if necessary. If the reference transcriptome is well annotated, researchers could analyze the biotype composition of the sample, which is indicative of the quality of the RNA purification step. For example, rRNA and small RNAs should not be present in regular polyA longRNA preparations [10, 19]. A number of R packages (such as NOISeq [19] or EDASeq [20]) provide useful plots for quality control of count data.

#### Reproducibility

The quality-control steps described above involve individual samples. In addition, it is also crucial to assess the global quality of the RNA-seq dataset by checking on the reproducibility among replicates and for possible batch effects. Reproducibility among technical replicates should be generally high (Spearman R^2^ > 0.9) [1], but no clear standard exists for biological replicates, as this depends on the heterogeneity of the experimental system. If gene expression differences exist among experimental conditions, it should be expected that biological replicates of the same condition will cluster together in a principal component analysis (PCA).

### Transcript identification

When a reference genome is available, RNA-seq analysis will normally involve the mapping of the reads onto the reference genome or transcriptome to infer which transcripts are expressed. Mapping solely to the reference transcriptome of a known species precludes the discovery of new, unannotated transcripts and focuses the analysis on quantification alone. By contrast, if the organism does not have a sequenced genome, then the analysis path is first to assemble reads into longer contigs and then to treat these contigs as the expressed transcriptome to which reads are mapped back again for quantification. In either case, read coverage can be used to quantify transcript expression level (Fig. 1b). A basic choice is whether transcript identification and quantification are done sequentially or simultaneously.

#### Alignment

**Fig. 2:**
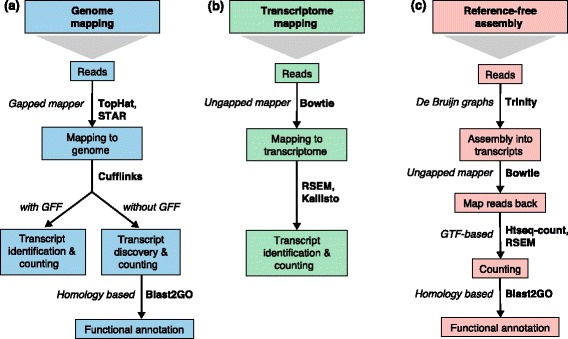
Read mapping and transcript identification strategies. Three basic strategies for regular RNA-seq analysis. **a** An annotated genome is available and reads are mapped to the genome with a gapped mapper. Next (novel) transcript discovery and quantification can proceed with or without an annotation file. Novel transcripts are then functionally annotated. **b** If no novel transcript discovery is needed, reads can be mapped to the reference transcriptome using an ungapped aligner. Transcript identification and quantification can occur simultaneously. **c** When no genome is available, reads need to be assembled first into contigs or transcripts. For quantification, reads are mapped back to the novel reference transcriptome and further analysis proceeds as in (**b**) followed by the functional annotation of the novel transcripts as in (**a**). Representative software that can be used at each analysis step are indicated in *bold text*. Abbreviations: *GFF* General Feature Format, *GTF* gene transfer format, *RSEM* RNA-Seq by Expectation Maximization

Two alternatives are possible when a reference sequence is available: mapping to the genome or mapping to the annotated transcriptome (Fig. 2a, b; Box 3). Regardless of whether a genome or transcriptome reference is used, reads may map uniquely (they can be assigned to only one position in the reference) or could be multi-mapped reads (multireads). Genomic multireads are primarily due to repetitive sequences or shared domains of paralogous genes. They normally account for a significant fraction of the mapping output when mapped onto the genome and should not be discarded. When the reference is the transcriptome, multi-mapping arises even more often because a read that would have been uniquely mapped on the genome would map equally well to all gene isoforms in the transcriptome that share the exon. In either case — genome or transcriptome mapping — transcript identification and quantification become important challenges for alternatively expressed genes.

#### Transcript discovery

Identifying novel transcripts using the short reads provided by Illumina technology is one of the most challenging tasks in RNA-seq. Short reads rarely span across several splice junctions and thus make it difficult to directly infer all full-length transcripts. In addition, it is difficult to identify transcription start and end sites [21], and tools such as GRIT [22] that incorporate other data such as 5’ ends from CAGE or RAMPAGE typically have a better chance of annotating the major expressed isoforms correctly. In any case, PE reads and higher coverage help to reconstruct lowly expressed transcripts, and replicates are essential to resolve false-positive calls (that is, mapping artifacts or contaminations) at the low end of signal detection. Several methods, such as Cufflinks [23], iReckon [24], SLIDE [25] and StringTie [26], incorporate existing annotations by adding them to the possible list of isoforms. Montebello [27] couples isoform discovery and quantification using a likelihood-based Monte Carlo algorithm to boost performance. Gene-finding tools such as Augustus [28] can incorporate RNA-seq data to better annotate protein-coding transcripts, but perform worse on non-coding transcripts [29]. In general, accurate transcript reconstruction from short reads is difficult, and methods typically show substantial disagreement [29].

#### De novo transcript reconstruction

When a reference genome is not available or is incomplete, RNA-seq reads can be assembled de novo (Fig. 2c) into a transcriptome using packages such as SOAPdenovo-Trans [30], Oases [31], Trans-ABySS [32] or Trinity [33]. In general, PE strand-specific sequencing and long reads are preferred because they are more informative [33]. Although it is impossible to assemble lowly expressed transcripts that lack enough coverage for a reliable assembly, too many reads are also problematic because they lead to potential misassembly and increased runtimes. Therefore, in silico reduction of the number of reads is recommended for deeply sequenced samples [33]. For comparative analyses across samples, it is advisable to combine all reads from multiple samples into a single input in order to obtain a consolidated set of contigs (transcripts), followed by mapping back of the short reads for expression estimation [33].

Either with a reference or de novo, the complete reconstruction of transcriptomes using short-read Illumina technology remains a challenging problem, and in many cases de novo assembly results in tens or hundreds of contigs accounting for fragmented transcripts. Emerging long-read technologies, such as SMRT from Pacific Biosciences, provide reads that are long enough to sequence complete transcripts for most genes and are a promising alternative that is discussed further in the “Outlook” section below.

### Transcript quantification

The most common application of RNA-seq is to estimate gene and transcript expression. This application is primarily based on the number of reads that map to each transcript sequence, although there are algorithms such as Sailfish that rely on *k*-mer counting in reads without the need for mapping [34]. The simplest approach to quantification is to aggregate raw counts of mapped reads using programs such as HTSeq-count [35] or featureCounts [36]. This gene-level (rather than transcript-level) quantification approach utilizes a gene transfer format (GTF) file [37] containing the genome coordinates of exons and genes, and often discard multireads. Raw read counts alone are not sufficient to compare expression levels among samples, as these values are affected by factors such as transcript length, total number of reads, and sequencing biases. The measure RPKM (reads per kilobase of exon model per million reads) [1] is a within-sample normalization method that will remove the feature-length and library-size effects. This measure and its subsequent derivatives FPKM (fragments per kilobase of exon model per million mapped reads), a within-sample normalized transcript expression measure analogous to RPKs, and TPM (transcripts per million) are the most frequently reported RNA-seq gene expression values. It should be noted that RPKM and FPKM are equivalent for SE reads and that FPKM can be converted into TPM using a simple formula [38]. The dichotomy of within-sample and between-sample comparisons has led to a lot of confusion in the literature. Correcting for gene length is not necessary when comparing changes in gene expression within the same gene across samples, but it is necessary for correctly ranking gene expression levels within the sample to account for the fact that longer genes accumulate more reads. Furthermore, programs such as Cufflinks that estimate gene length from the data can find significant differences in gene length between samples that cannot be ignored. TPMs, which effectively normalize for the differences in composition of the transcripts in the denominator rather than simply dividing by the number of reads in the library, are considered more comparable between samples of different origins and composition but can still suffer some biases. These must be addressed with normalization techniques such as TMM.

Several sophisticated algorithms have been developed to estimate transcript-level expression by tackling the problem of related transcripts’ sharing most of their reads. Cufflinks [39] estimates transcript expression from a mapping to the genome obtained from mappers such as TopHat using an expectation-maximization approach that estimates transcript abundances. This approach takes into account biases such as the non-uniform read distribution along the gene length. Cufflinks was designed to take advantage of PE reads, and may use GTF information to identify expressed transcripts, or can infer transcripts de novo from the mapping data alone. Algorithms that quantify expression from transcriptome mappings include RSEM (RNA-Seq by Expectation Maximization) [40], eXpress [41], Sailfish [35] and kallisto [42] among others. These methods allocate multi-mapping reads among transcript and output within-sample normalized values corrected for sequencing biases [35, 41, 43]. Additionally, the RSEM algorithm uses an expectation maximization approach that returns TPM values [40]. NURD [44] provides an efficient way of estimating transcript expression from SE reads with a low memory and computing cost.

### Differential gene expression analysis

Differential expression analysis (Fig. 1b) requires that gene expression values should be compared among samples. RPKM, FPKM, and TPM normalize away the most important factor for comparing samples, which is sequencing depth, whether directly or by accounting for the number of transcripts, which can differ significantly between samples. These approaches rely on normalizing methods that are based on total or effective counts, and tend to perform poorly when samples have heterogeneous transcript distributions, that is, when highly and differentially expressed features can skew the count distribution [45, 46]. Normalization methods that take this into account are TMM [47], DESeq [48], PoissonSeq [49] and UpperQuartile [45], which ignore highly variable and/or highly expressed features. Additional factors that interfere with intra-sample comparisons include changes in transcript length across samples or conditions [50], positional biases in coverage along the transcript (which are accounted for in Cufflinks), average fragment size [43], and the GC contents of genes (corrected in the EDAseq package [21]). The NOISeq R package [20] contains a wide variety of diagnostic plots to identify sources of biases in RNA-seq data and to apply appropriate normalization procedures in each case. Finally, despite these sample-specific normalization methods, batch effects may still be present in the data. These effects can be minimized by appropriate experimental design [51] or, alternatively, removed by batch-correction methods such as COMBAT [52] or ARSyN [20, 53]. These approaches, although initially developed for microarray data, have been shown to work well with normalized RNA-seq data (STATegra project, unpublished).

As RNA-seq quantification is based on read counts that are absolutely or probabilistically assigned to transcripts, the first approaches to compute differential expression used discrete probability distributions, such as the Poisson or negative binomial [48, 54]. The negative binomial distribution (also known as the gamma-Poisson distribution) is a generalization of the Poisson distribution, allowing for additional variance (called overdispersion) beyond the variance expected from randomly sampling from a pool of molecules that are characteristic of RNA-seq data. However, the use of discrete distributions is not required for accurate analysis of differential expression as long as the sampling variance of small read counts is taken into account (most important for experiments with small numbers of replicates). Methods for transforming normalized counts of RNA-seq reads while learning the variance structure of the data have been shown to perform well in comparison to the discrete distribution approaches described above [55, 56]. Moreover, after extensive normalization (including TMM and batch removal), the data might have lost their discrete nature and be more akin to a continuous distribution.

Some methods, such as the popular edgeR [57], take as input raw read counts and introduce possible bias sources into the statistical model to perform an integrated normalization as well as a differential expression analysis. In other methods, the differential expression requires the data to be previously normalized to remove all possible biases. DESeq2, like edgeR, uses the negative binomial as the reference distribution and provides its own normalization approach [48, 58]. baySeq [59] and EBSeq [60] are Bayesian approaches, also based on the negative binomial model, that define a collection of models to describe the differences among experimental groups and to compute the posterior probability of each one of them for each gene. Other approaches include data transformation methods that take into account the sampling variance of small read counts and create discrete gene expression distributions that can be analyzed by regular linear models [55]. Finally, non-parametric approaches such as NOISeq [10] or SAMseq [61] make minimal assumptions about the data and estimate the null distribution for inferential analysis from the actual data alone. For small-scale studies that compare two samples with no or few replicates, the estimation of the negative binomial distribution can be noisy. In such cases, simpler methods based on the Poisson distribution, such as DEGseq [62], or on empirical distributions (NOISeq [10]) can be an alternative, although it should be strongly stressed that, in the absence of biological replication, no population inference can be made and hence any *p* value calculation is invalid. Methods that analyze RNA-seq data without replicates therefore only have exploratory value. Considering the drop in price of sequencing, we recommend that RNA-seq experiments have a minimum of three biological replicates when sample availability is not limiting to allow all of the differential expression methods to leverage reproducibility between replicates.

Recent independent comparison studies have demonstrated that the choice of the method (or even the version of a software package) can markedly affect the outcome of the analysis and that no single method is likely to perform favorably for all datasets [56, 63, 64] (Box 4). We therefore recommend thoroughly documenting the settings and version numbers of programs used and considering the repetition of important analyses using more than one package.

### Alternative splicing analysis

Transcript-level differential expression analysis can potentially detect changes in the expression of transcript isoforms from the same gene, and specific algorithms for alternative splicing-focused analysis using RNA-seq have been proposed. These methods fall into two major categories. The first approach integrates isoform expression estimation with the detection of differential expression to reveal changes in the proportion of each isoform within the total gene expression. One such early method, BASIS, used a hierarchical Bayesian model to directly infer differentially expressed transcript isoforms [65]. CuffDiff2 estimates isoform expression first and then compares their differences. By integrating the two steps, the uncertainty in the first step is taken into consideration when performing the statistical analysis to look for differential isoform expression [66]. The flow difference metric (FDM) uses aligned cumulative transcript graphs from mapped exon reads and junction reads to infer isoforms and the Jensen-Shannon divergence to measure the difference [67]. Recently, Shi and Jiang [68] proposed a new method, rSeqDiff, that uses a hierarchical likelihood ratio test to detect differential gene expression without splicing change and differential isoform expression simultaneously. All these approaches are generally hampered by the intrinsic limitations of short-read sequencing for accurate identification at the isoform level, as discussed in the RNA-seq Genome Annotation Assessment Project paper [30].

The so-called ‘exon-based’ approach skips the estimation of isoform expression and detects signals of alternative splicing by comparing the distributions of reads on exons and junctions of the genes between the compared samples. This approach is based on the premise that differences in isoform expression can be tracked in the signals of exons and their junctions. DEXseq [69] and DSGSeq [70] adopt a similar idea to detect differentially spliced genes by testing for significant differences in read counts on exons (and junctions) of the genes. rMATS detects differential usage of exons by comparing exon-inclusion levels defined with junction reads [71]. rDiff detects differential isoform expression by comparing read counts on alternative regions of the gene, either with or without annotated alternative isoforms [72]. DiffSplice uses alignment graphs to identify alternative splicing modules (ASMs) and identifies differential splicing using signals of the ASMs [73]. The advantage of exon or junction methods is their greater accuracy in identifying individual alternative splicing events. Exon-based methods are appropriate if the focus of the study is not on whole isoforms but on the inclusion and exclusion of specific exons and the functional protein domains (or regulatory features, in case of untranslated region exons) that they contain.

### Visualization

Visualization of RNA-seq data (Fig. 1c) is, in general terms, similar to that of any other type of genomic sequencing data, and it can be done at the level of reads (using ReadXplorer [74], for example) or at the level of processed coverage (read pileup), unnormalized (for example, total count) or normalized, using genome browsers such as the UCSC browser [75], Integrative Genomics Viewer (IGV) [76] (Figure S1a in Additional file 1), Genome Maps [77], or Savant [78]. Some visualization tools are specifically designed for visualizing multiple RNA-seq samples, such as RNAseqViewer [79], which provides flexible ways to display the read abundances on exons, transcripts and junctions. Introns can be hidden to better display signals on the exons, and the heatmaps can help the visual comparison of signals on multiple samples (Figure S1b, c in Additional file 1). However, RNAseqViewer is slower than IGV.

Some of the software packages for differential gene expression analysis (such as DESeq2 or DEXseq in Bioconductor) have functions to enable the visualization of results, whereas others have been developed for visualization-exclusive purposes, such as CummeRbund (for CuffDiff [66]) or Sashimi plots, which can be used to visualize differentially spliced exons [80]. The advantage of Sashimi plots is that their display of junction reads is more intuitive and aesthetically pleasing when the number of samples is small (Figure S1d in Additional file 1). Sashimi, structure, and hive plots for splicing quantitative trait loci (sQTL) can be obtained using SplicePlot [81]. Splice graphs can be produced using SpliceSeq [82], and SplicingViewer [83] plots splice junctions and alternative splicing events. TraV [84] is a visualization tool that integrates data analysis, but its analytical methods are not applicable to large genomes.

Owing to the complexity of transcriptomes, efficient display of multiple layers of information is still a challenge. All of the tools are evolving rapidly and we can expect more comprehensive tools with desirable features to be available soon. Nevertheless, the existing tools are of great value for exploring results for individual genes of biological interest to assess whether particular analyses’ results can withstand detailed scrutiny or to reveal potential complications caused by artifacts, such as 3’ biases or complicated transcript structures. Users should visualize changes in read coverage for genes that are deemed important or interesting on the basis of their analysis results to evaluate the robustness of their conclusions.

### Gene fusion discovery

The discovery of fused genes that can arise from chromosomal rearrangements is analogous to novel isoform discovery, with the added challenge of a much larger search space as we can no longer assume that the transcript segments are co-linear on a single chromosome. Artifacts are common even using state-of-the-art tools, which necessitates post-processing using heuristic filters [85]. Artifacts primarily result from misalignment of read sequences due to polymorphisms, homology, and sequencing errors. Families of homologous genes, and highly polymorphic genes such as the HLA genes, produce reads that cannot be easily mapped uniquely to their location of origin in the reference genome. For genes with very high expression, the small but non-negligible sequencing error rate of RNA-seq will produce reads that map incorrectly to homologous loci. Filtering highly polymorphic genes and pairs of homologous genes is recommended [86, 87]. Also recommended is the filtering of highly expressed genes that are unlikely to be involved in gene fusions, such as ribosomal RNA [86]. Finally, a low ratio of chimeric to wild-type reads in the vicinity of the fusion boundary may indicate spurious mis-mapping of reads from a highly expressed gene (the transcript allele fraction described by Yoshihara et al. [87]).

Given successful prediction of chimeric sequences, the next step is the prioritization of gene fusions that have biological impact over more expected forms of genomic variation. Examples of expected variation include immunoglobulin (IG) rearrangements in tumor samples infiltrated by immune cells, transiently expressed transposons and nuclear mitochondrial DNA, and read-through chimeras produced by co-transcription of adjacent genes [88]. Care must be taken with filtering in order not to lose events of interest. For example, removing all fusions involving an IG gene may remove real IG fusions in lymphomas and other blood disorders; filtering fusions for which both genes are from the IG locus is preferred [88]. Transiently expressed genomic breakpoint sequences that are associated with real gene fusions often overlap transposons; these should be filtered unless they are associated with additional fusion isoforms from the same gene pair [89]. Read-through chimeras are easily identified as predictions involving alternative splicing between adjacent genes. Where possible, fusions should be filtered by their presence in a set of control datasets [87]. When control datasets are not available, artifacts can be identified by their presence in a large number of unrelated datasets, after excluding the possibility that they represent true recurrent fusions [90, 91].

Strong fusion-sequence predictions are characterized by distinct subsequences that each align with high specificity to one of the fused genes. As alignment specificity is highly correlated with sequence length, a strong prediction sequence is longer, with longer subsequences from each gene. Longer reads and larger insert sizes produce longer predicted sequences; thus, we recommend PE RNA-seq data with larger insert size over SE datasets or datasets with short insert size. Another indicator of prediction strength is splicing. For most known fusions, the genomic breakpoint is located in an intron of each gene [92] and the fusion boundary coincides with a splice site within each gene. Furthermore, fusion isoforms generally follow the splicing patterns of wild-type genes. Thus, high confidence predictions have fusion boundaries coincident with exon boundaries and exons matching wild-type exons [91]. Fusion discovery tools often incorporate some of the aforementioned ideas to rank fusion predictions [93, 94], though most studies apply additional custom heuristic filters to produce a list of high-quality fusion candidates [90, 91, 95].

### Small RNAs

Next-generation sequencing represents an increasingly popular method to address questions concerning the biological roles of small RNAs (sRNAs). sRNAs are usually 18–34 nucleotides in length, and they include miRNAs, short-interfering RNAs (siRNAs), PIWI-interacting RNAs (piRNAs), and other classes of regulatory molecules. sRNA-seq libraries are rarely sequenced as deeply as regular RNA-seq libraries because of a lack of complexity, with a typical range of 2–10 million reads. Bioinformatics analysis of sRNA-seq data differs from standard RNA-seq protocols (Fig. 1c). Ligated adaptor sequences are first trimmed and the resulting read-length distribution is computed. In animals, there are usually peaks for 22 and 23 nucleotides, whereas in plants there are peaks for 21- and 24-nucleotide redundant reads. For instance, miRTools 2.0 [96], a tool for prediction and profiling of sRNA species, uses by default reads that are 18–30 bases long. The threshold value depends on the application, and in case of miRNAs is usually in the range of 19–25 nucleotides.

As in standard RNA-seq, sRNA reads must then be aligned to a reference genome or transcriptome sequences using standard tools, such as Bowtie2 [97], STAR [15], or Burrows-Wheeler Aligner (BWA) [98]. There are, however, some aligners (such as PatMaN [99] and MicroRazerS [100]) that have been designed to map short sequences with preset parameter value ranges suited for optimal alignment of short reads. The mapping itself may be performed with or without mismatches, the latter being used more commonly. In addition, reads that map beyond a predetermined set number of locations may be removed as putatively originating from repetitive elements. In the case of miRNAs, usually 5–20 distinct mappings per genome are allowed. sRNA reads are then simply counted to obtain expression values. However, users should also verify that their sRNA reads are not significantly contaminated by degraded mRNA, for example, by checking whether a miRNA library shows unexpected read coverage over the body of highly expressed genes such as *GAPDH* or *ACTB*.

Further analysis steps include comparison with known sRNAs and de novo identification of sRNAs. There are class-specific tools for this purpose, such as miRDeep [101] and miRDeep-P [102] for animal and plant miRNAs, respectively, or the trans-acting siRNA prediction tool at the UEA sRNA Workbench [103]. Tools such as miRTools 2.0 [96], ShortStack [104], and iMir [105] also exist for comprehensive annotation of sRNA libraries and for identification of diverse classes of sRNAs.

### Functional profiling with RNA-seq

The last step in a standard transcriptomics study (Fig. 1b) is often the characterization of the molecular functions or pathways in which differentially expressed genes (DEGs) are involved. The two main approaches to functional characterization that were developed first for microarray technology are (a) comparing a list of DEGs against the rest of the genome for overrepresented functions, and (b) gene set enrichment analysis (GSEA), which is based on ranking the transcriptome according to a measurement of differential expression. RNA-seq biases such as gene length complicate the direct applications of these methods for count data and hence RNA-seq-specific tools have been proposed. For example, GOseq [106] estimates a bias effect (such as gene length) on differential expression results and adapts the traditional hypergeometric statistic used in the functional enrichment test to account for this bias. Similarly, the Gene Set Variation Analysis (GSVA) [107] or SeqGSEA [108] packages also combine splicing and implement enrichment analyses similar to GSEA.

Functional analysis requires the availability of sufficient functional annotation data for the transcriptome under study. Resources such as Gene Ontology [109], Bioconductor [110], DAVID [111, 112] or Babelomics [113] contain annotation data for most model species. However, novel transcripts discovered during de novo transcriptome assembly or reconstruction would lack at least some functional information and therefore annotation is necessary for functional profiling of those results. Protein-coding transcripts can be functionally annotated using orthology by searching for similar sequences in protein databases such as SwissProt [114] and in databases that contain conserved protein domains such as Pfam [115] and InterPro [116]. The use of standard vocabularies such as the Gene Ontology (GO) allows for some exchangeability of functional information across orthologs. Popular tools such as Blast2GO [117] allow massive annotation of complete transcriptome datasets against a variety of databases and controlled vocabularies. Typically, between 50 and 80 % of the transcripts reconstructed from RNA-seq data can be annotated with functional terms in this way. However, RNA-seq data also reveal that an important fraction of the transcriptome is lacking protein-coding potential. The functional annotation of these long non-coding RNAs is more challenging as their conservation is often less pronounced than that of protein-coding genes. The Rfam database [118] contains most well-characterized RNA families, such as ribosomal or transfer RNAs, while mirBase [119] or Miranda [120] are specialized in miRNAs. These resources can be used for similarity-based annotation of short non-coding RNAs, but no standard functional annotation procedures are available yet for other RNA types such as the long non-coding RNAs.

## Integration with other data types

The integration of RNA-seq data with other types of genome-wide data (Fig. 1c) allows us to connect the regulation of gene expression with specific aspects of molecular physiology and functional genomics. Integrative analyses that incorporate RNA-seq data as the primary gene expression readout that is compared with other genomic experiments are becoming increasingly prevalent. Below, we discuss some of the additional challenges posed by such analyses.

### DNA sequencing

The combination of RNA and DNA sequencing can be used for several purposes, such as single nucleotide polymorphism (SNP) discovery, RNA-editing analyses, or expression quantitative trait loci (eQTL) mapping. In a typical eQTL experiment, genotype and transcriptome profiles are obtained from the same tissue type across a relatively large number of individuals (>50) and correlations between genotype and expression levels are then detected. These associations can unravel the genetic basis of complex traits such as height [121], disease susceptibility [122] or even features of genome architecture [123, 124]. Large eQTL studies have shown that genetic variation affects the expression of most genes [125–128].

RNA-seq has two major advantages over array-based technologies for detecting eQTLs. First, it can identify variants that affect transcript processing. Second, reads that overlap heterozygous SNPs can be mapped to maternal and paternal chromosomes, enabling quantification of allele-specific expression within an individual [129]. Allele-specific signals provide additional information about a genetic effect on transcription, and a number of computational methods have recently become available that leverage these signals to boost power for association mapping [130–132]. One challenge of this approach is the computational burden, as billions of gene–SNP associations need to be tested; bootstrapping or permutation-based approaches [133] are frequently used [134, 135]. Many studies have focused on testing only SNPs in the *cis* region surrounding the gene in question, and computationally efficient approaches have been developed recently to allow extremely swift mapping of eQTLs genome-wide [136]. Moreover, the combination of RNA-seq and re-sequencing can be used both to remove false positives when inferring fusion genes [88] and to analyze copy number alterations [137].

### DNA methylation

Pairwise DNA-methylation and RNA-seq integration, for the most part, has consisted of the analysis of the correlation between DEGs and methylation patterns [138–140]. General linear models [141–143], logistic regression models [143] and empirical Bayes model [144] have been attempted among other modeling approaches. The statistically significant correlations that were observed, however, accounted for relatively small effects. An interesting shift away from focusing on individual gene–CpG methylation correlations is to use a network-interaction-based approach to analyze RNA-seq in relation to DNA methylation. This approach identifies one or more sets of genes (also called modules) that have coordinated differential expression and differential methylation [145].

### Chromatin features

The combination of RNA-seq and transcription factor (TF) chromatin immunoprecipitation sequencing (ChIP-seq) data can be used to remove false positives in ChIP-seq analysis and to suggest the activating or repressive effect of a TF on its target genes. For example, BETA [146] uses differential gene expression in combination with peaks from ChIP-seq experiments to call TF targets. In addition, ChIP-seq experiments involving histone modifications have been used to understand the general role of these epigenomic changes on gene expression [147, 148]. Other RNA-ChIP-sequencing integrative approaches are reviewed in [149]. Integration of open chromatin data such as that from FAIRE-seq and DNase-seq with RNA-seq has mostly been limited to verifying the expression status of genes that overlap a region of interest [150]. DNase-seq can be used for genome-wide footprinting of DNA-binding factors, and this in combination with the actual expression of genes can be used to infer active transcriptional networks [150].

### MicroRNAs

Integration of RNA-seq and miRNA-seq data has the potential to unravel the regulatory effects of miRNAs on transcript steady-state levels. This analysis is challenging, however, because of the very noisy nature of miRNA target predictions, which hampers analyses based on correlations between miRNAs and their target genes. Associations might be found in databases such as mirWalk [151] and miRBase [152] that offer target prediction according to various algorithms. Tools such as CORNA [153], MMIA [154, 155], MAGIA [156], and SePIA [157] refine predictions by testing for significant associations between genes, miRNAs, pathways and GO terms, or by testing the relatedness or anticorrelation of the expression profiles of both the target genes and the associated miRNAs. In general, we recommend using miRNA–mRNA associations that are predicted by several algorithms. For example, in mouse, we found that requiring miRNA–mRNA association in five databases resulted in about 50 target mRNA predictions per miRNA (STATegra observations).

### Proteomics and metabolomics

Integration of RNA-seq with proteomics is controversial because the two measurements show generally low correlation (~0.40 [158, 159]). Nevertheless, pairwise integration of proteomics and RNA-seq can be used to identify novel isoforms. Unreported peptides can be predicted from RNA-seq data and then used to complement databases normally queried in mass spectrometry as done by Low et al. [160]. Furthermore, post-translational editing events may be identified if peptides that are present in the mass spectrometry analysis are absent from the expressed genes of the RNA-seq dataset. Integration of transcriptomics with metabolomics data has been used to identify pathways that are regulated at both the gene expression and the metabolite level, and tools are available that visualize results within the pathway context (MassTRIX [161], Paintomics [162], VANTED v2 [163], and SteinerNet [164]).

### Integration and visualization of multiple data types

Integration of more than two genomic data types is still at its infancy and not yet extensively applied to functional sequencing techniques, but there are already some tools that combine several data types. SNMNMF [165] and PIMiM [166] combine mRNA and miRNA expression data with protein–protein, DNA–protein, and miRNA–mRNA interaction networks to identify miRNA–gene regulatory modules. MONA [167] combines different levels of functional genomics data, including mRNA, miRNA, DNA methylation, and proteomics data to discover altered biological functions in the samples being studied. Paintomics can integrate any type of functional genomics data into pathway analysis, provided that the features can be mapped onto genes or metabolites [162]. 3Omics [168] integrates transcriptomics, metabolomics and proteomics data into regulatory networks.

In all cases, integration of different datasets is rarely straightforward because each data type is analyzed separately with its own tailored algorithms that yield results in different formats. Tools that facilitate format conversions and the extraction of relevant results can help; examples of such workflow construction software packages include Anduril [169], Galaxy [170] and Chipster [171]. Anduril was developed for building complex pipelines with large datasets that require automated parallelization. The strength of Galaxy and Chipster is their usability; visualization is a key component of their design. Simultaneous or integrative visualization of the data in a genome browser is extremely useful for both data exploration and interpretation of results. Browsers can display in tandem mappings from most next-generation sequencing technologies, while adding custom tracks such as gene annotation, nucleotide variation or ENCODE datasets. For proteomics integration, the PG Nexus pipeline [172] converts mass spectrometry data to mappings that are co-visualized with RNA-seq alignments.

## Outlook

RNA-seq has become the standard method for transcriptome analysis, but the technology and tools are continuing to evolve. It should be noted that the agreement between results obtained from different tools is still unsatisfactory and that results are affected by parameter settings, especially for genes that are expressed at low levels. The two major highlights in the current application of RNA-seq are the construction of transcriptomes from small amounts of starting materials and better transcript identification from longer reads. The state of the art in both of these areas is changing rapidly, but we will briefly outline what can be done now and what can be expected in the near future.

### Single-cell RNA-seq

Single-cell RNA-seq (scRNA-seq) is one of the newest and most active fields of RNA-seq with its unique set of opportunities and challenges. Newer protocols such as Smart-seq [173] and Smart-seq2 [174] have enabled us to work from very small amounts of starting mRNA that, with proper amplification, can be obtained from just a single cell. The resulting single-cell libraries enable the identification of new, uncharacterized cell types in tissues. They also make it possible to measure a fascinating phenomenon in molecular biology, the stochasticity of gene expression in otherwise identical cells within a defined population. In this context, single cell studies are meaningful only when a set of individual cell libraries are compared with the cell population, with the aim of identifying subgroups of multiple cells with distinct combinations of expressed genes. Differences may be due to naturally occurring factors such as stage of the cell cycle, or may reflect rare cell types such as cancer stem cells. Recent rapid progress in methodologies for single-cell preparation, including the availability of single-cell platforms such as the Fluidigm C1 [8], has increased the number of individual cells analyzed from a handful to 50–90 per condition up to 800 cells at a time. Other methods, such as DROP-seq [175], can profile more than 10,000 cells at a time. This increased number of single-cell libraries in each experiment directly allows for the identification of smaller subgroups within the population.

The small amount of starting material and the PCR amplification limit the depth to which single-cell libraries can be sequenced productively, often to less than a million reads. Deeper sequencing for scRNA-seq will do little to improve quantification as the number of individual mRNA molecules in a cell is small (in the order of 100–300,000 transcripts) and only a fraction of them are successfully reverse-transcribed to cDNA [8, 176]; but deeper sequencing is potentially useful for discovering and measuring allele-specific expression, as additional reads could provide useful evidence.

Single-cell transcriptomes typically include about 3000–8000 expressed genes, which is far fewer than are counted in the transcriptomes of the corresponding pooled populations. The challenge is to distinguish the technical noise that results from a lack of sensitivity at the single-molecule level [173] (where capture rates of around 10–50 % result in the frequent loss of the most lowly expressed transcripts) from true biological noise where a transcript might not be transcribed and present in the cell for a certain amount of time while the protein is still present. The inclusion of added reference transcripts and the use of unique molecule identifiers (UMIs) have been applied to overcome amplification bias and to improve gene quantification [177, 178]. Methods that can quantify gene-level technical variation allow us to focus on biological variation that is likely to be of interest [179]. Typical quality-control steps involve setting aside libraries that contain few reads, libraries that have a low mapping rate, and libraries that have zero expression levels for housekeeping genes, such as *GAPDH* and *ACTB*, that are expected to be expressed at a detectable level.

Depending on the chosen single-cell protocol and the aims of the experiment, different bulk RNA-seq pipelines and tools can be used for different stages of the analysis as reviewed by Stegle et al. [180]. Single-cell libraries are typically analyzed by mapping to a reference transcriptome (using a program such as RSEM) without any attempt at new transcript discovery, although at least one package maps to the genome (Monocle [181]). While mapping onto the genome does result in a higher overall read-mapping rate, studies that are focused on gene expression alone with fewer reads per cell tend to use mapping to the reference transcriptome for the sake of simplicity. Other single-cell methods have been developed to measure single-cell DNA methylation [182] and single-cell open chromatin using ATAC-seq [183, 184]. At present, we can measure only one functional genomic data-type at a time in the same single cell, but we can expect that in the near future we will be able to recover the transcriptome of a single cell simultaneously with additional functional data.

### Long-read sequencing

The major limitation of short-read RNA-seq is the difficulty in accurately reconstructing expressed full-length transcripts from the assembly of reads. This is particularly complicated in complex transcriptomes, where different but highly similar isoforms of the same gene are expressed, and for genes that have many exons and possible alternative promoters or 3’ ends. Long-read technologies, such as Pacific-Biosciences (PacBio) SMRT and Oxford Nanopore, that were initially applied to genome sequencing are now being used for transcriptomics and have the potential to overcome this assembly problem. Long-read sequencing provides amplification-free, single-molecule sequencing of cDNAs that enables recovery of full-length transcripts without the need for an assembly step. PacBio adds adapters to the cDNA molecule and creates a circularized structure that can be sequenced with multiple passes within one single long read. The Nanopore GridION system can directly sequence RNA strands by using RNA processive enzymes and RNA-specific bases. Another interesting technology was previously known as Moleculo (now Illumina’s TruSeq synthetic long-read technology), where Illumina library preparation is multiplexed and restricted to a limited number of long DNA molecules that are separately bar-coded and pooled back for sequencing. As one barcode corresponds to a limited number of molecules, assembly is greatly simplified and unambiguous reconstruction to long contigs is possible. This approach has recently been published for RNA-seq analysis [185].

PacBio RNA-seq is the long-read approach with the most publications to date. The technology has proven useful for unraveling isoform diversity at complex loci [186], and for determining allele-specific expression from single reads [187]. Nevertheless, long-read sequencing has its own set of limitations, such as a still high error rate that limits de novo transcript identifications and forces the technology to leverage the reference genome [188]. Moreover, the relatively low throughput of SMRT cells hampers the quantification of transcript expression. These two limitations can be addressed by matching PacBio experiments with regular, short-read RNA-seq. The accurate and abundant Illumina reads can be used both to correct long-read sequencing errors and to quantify transcript levels [189]. Updates in PacBio chemistry are increasing sequencing lengths to produce reads with a sufficient number of passes over the cDNA molecule to autocorrect sequencing errors. This will eventually improve sequencing accuracy and allow for genome-free determination of isoform-resolved transcriptomes.

## Box 1. Number of replicates

Three factors determine the number of replicates required in a RNA-seq experiment. The first factor is the variability in the measurements, which is influenced by the technical noise and the biological variation. While reproducibility in RNA-seq is usually high at the level of sequencing [1, 45], other steps such as RNA extraction and library preparation are noisier and may introduce biases in the data that can be minimized by adopting good experimental procedures (Box 2). Biological variability is particular to each experimental system and is harder to control [189]. Nevertheless, biological replication is required if inference on the population is to be made, with three replicates being the minimum for any inferential analysis. For a proper statistical power analysis, estimates of the within-group variance and gene expression levels are required. This information is typically not available beforehand but can be obtained from similar experiments. The exact power will depend on the method used for differential expression analysis, and software packages exist that provide a theoretical estimate of power over a range of variables, given the within-group variance of the samples, which is intrinsic to the experiment [190, 191]. Table 1 shows an example of statistical power calculations over a range of fold-changes (or effect sizes) and number of replicates in a human blood RNA-seq sample sequenced at 30 million mapped reads. It should be noted that these estimates apply to the average gene expression level, but as dynamic ranges in RNA-seq data are large, the probability that highly expressed genes will be detected as differentially expressed is greater than that for low-count genes [192]. For methods that return a false discovery rate (FDR), the proportion of genes that are highly expressed out of the total set of genes being tested will also influence the power of detection after multiple testing correction [193]. Filtering out genes that are expressed at low levels prior to differential expression analysis reduces the severity of the correction and may improve the power of detection [20]. Increasing sequencing depth also can improve statistical power for lowly expressed genes [10, 194], and for any given sample there exists a level of sequencing at which power improvement is best achieved by increasing the number of replicates [195]. Tools such as Scotty are available to calculate the best trade-off between sequencing depth and replicate number given some budgetary constraints [191].

**Table 1:** Statistical power to detect differential expression varies with effect size, sequencing depth and number of replicates

	Replicates per group		
	3	5	10
Effect size (fold change)			
1.25	17 %	25 %	44 %
1.5	43 %	64 %	91 %
2	87 %	98 %	100 %
Sequencing depth (millions of reads)			
3	19 %	29 %	52 %
10	33 %	51 %	80 %
15	38 %	57 %	85 %

Example of calculations for the probability of detecting differential expression in a single test at a significance level of 5 %, for a two-group comparison using a Negative Binomial model, as computed by the RNASeqPower package of Hart et al. [190]. For a fixed within-group variance (package default value), the statistical power increases with the difference between the two groups (effect size), the sequencing depth, and the number of replicates per group. This table shows the statistical power for a gene with 70 aligned reads, which was the median coverage for a protein-coding gene for one whole-blood RNA-seq sample with 30 million aligned reads from the GTEx Project [214]

## Box 2. Experiment execution choices

RNA-seq library preparation and sequencing procedures include a number of steps (RNA fragmentation, cDNA synthesis, adapter ligation, PCR amplification, bar-coding, and lane loading) that might introduce biases into the resulting data [196]. Including exogenous reference transcripts (‘spike-ins’) is useful both for quality control [1, 197] and for library-size normalization [198]. For bias minimization, we recommend following the suggestions made by Van Dijk et al. [199], such as the use of adapters with random nucleotides at the extremities or the use of chemical-based fragmentation instead of RNase III-based fragmentation. If the RNA-seq experiment is large and samples have to be processed in different batches and/or Illumina runs, caution should be taken to randomize samples across library preparation batches and lanes so as to avoid technical factors becoming confounded with experimental factors. Another option, when samples are individually barcoded and multiple Illumina lanes are needed to achieve the desired sequencing depth, is to include all samples in each lane, which would minimize any possible lane effect.

## Box 3. Mapping to a reference

Mapping to a reference genome allows for the identification of novel genes or transcripts, and requires the use of a gapped or spliced mapper as reads may span splice junctions. The challenge is to identify splice junctions correctly, especially when sequencing errors or differences with the reference exist or when non-canonical junctions and fusion transcripts are sought. One of the most popular RNA-seq mappers, TopHat, follows a two-step strategy in which unspliced reads are first mapped to locate exons, then unmapped reads are split and aligned independently to identify exon junctions [200, 201]. Several other mappers exist that are optimized to identify SNPs or indels (GSNAP [202], PALMapper [203] MapSplice [204]), detect non-canonical splice junctions (STAR [15], MapSplice [204]), achieve ultra-fast mapping (GEM [205]) or map long-reads (STAR [15]). Important parameters to consider during mapping are the strandedness of the RNA-seq library, the number of mismatches to accept, the length and type of reads (SE or PE), and the length of sequenced fragments. In addition, existing gene models can be leveraged by supplying an annotation file to some read mapper in order to map exon coordinates accurately and to help in identifying splicing events. The choice of gene model can also have a strong impact on the quantification and differential expression analysis [206]. We refer the reader to [30] for a comprehensive comparison of RNA-seq mappers. If the transcriptome annotation is comprehensive (for example, in mouse or human), researchers may choose to map directly to a Fasta-format file of all transcript sequences for all genes of interests. In this case, no gapped alignment is needed and unspliced mappers such as Bowtie [207] can be used (Fig. 2b). Mapping to the transcriptome is generally faster but does not allow de novo transcript discovery.

## Box 4. Comparison of software tools for detecting differential gene and transcript expression

Many statistical methods are available for detecting differential gene or transcript expression from RNA-seq data, and a major practical challenge is how to choose the most suitable tool for a particular data analysis job. Most comparison studies have focused on simulated datasets [56, 208, 209] or on samples to which exogenous RNA (‘spike-in’) has been added in known quantities [63, 196]. This enables a direct assessment of the sensitivity and specificity of the methods as well as their FDR control. As simulations typically rely on specific statistical distributions or on limited experimental datasets and as spike-in datasets represent only technical replicates with minimal variation, comparisons using simulated datasets have been complemented with more practical comparisons in real datasets with true biological replicates [64, 210, 211].

As yet, no clear consensus has been reached regarding the best practices and the field is continuing to evolve rapidly. However, some common findings have been made in multiple comparison studies and in different study settings. First, specific caution is needed with all the methods when the number of replicate samples is very small or for genes that are expressed at very low levels [55, 64, 209]. Among the tools, limma has been shown to perform well under many circumstances and it is also the fastest to run [56, 63, 64]. DESeq and edgeR perform similarly in ranking genes but are often relatively conservative or too liberal, respectively, in controlling FDR [63, 209, 210]. SAMseq performs well in terms of FDR but presents an acceptable sensitivity when the number of replicates is relatively high, at least 10 [20, 55, 209]. NOISeq and NOISeqBIO (the adaptation of NOISeq for biological replication) are more efficient in avoiding false positive calls at the cost of some sensitivity but perform well with different numbers of replicates [10, 20, 212]. Cuffdiff and Cuffdiff2 have performed surprisingly poorly in the comparisons [56, 63]. This probably reflects the fact that detecting differential expression at the transcript level remains challenging and involves uncertainties in assigning the reads to alternative isoforms. In a recent comparison, BitSeq compared favorably to other transcript-level packages such as Cuffdiff2 [196]. Besides the actual performance, other issues affecting the choice of the tool include ease of installation and use, computational requirements, and quality of documentation and instructions. Finally, an important consideration when choosing an analysis method is the experimental design. While some of the differential expression tools can only perform a pair-wise comparison, others such as edgeR [57], limma-voom [55], DESeq [48], DESeq2 [58], and maSigPro [213] can perform multiple comparisons, include different covariates or analyze time-series data.

## Additional file

Additional file 1: Figure S1. Screenshots of RNA-seq data visualization. **a** Integrative Genomics Viewer (IGV) [77] display of a gene detected as differentially expressed between the two groups of samples by DEGseq [62]. The bottom track in the right panel is the gene annotation. The tracks are five samples from each group. **b** RNAseqViewer [80] display of the same data as in (**a**). **c** RNAseqViewer heatmap display of a gene detected as differentially spliced between two groups by both DSGSeq [70] and DEXSeq [69]. Introns are hidden in the display to emphasize the signals on the exons. **d** MISO [81] display of another gene detected as differentially spliced, with junction reads illustrated. (PDF 1152 kb)

## Abbreviations

ASM: Alternative splicing module
ChIP-seq: Chromatin immunoprecipitation sequencing
DEG: Differentially expressed genes
eQTL: Expression quantitative loci
FDR: False discovery rate
FPKM: Fragments per kilobase of exon model per million mapped reads
GO: Gene Ontology
GSEA: Gene set enrichment analysis
GTF: Gene transfer format
IG: Immunoglobulin
IGV: Integrative Genomics Viewer
miRNA: MicroRNA
mRNA: Messenger RNA
PCA: Principal component analysis
PE read: Paired-end read
RNA-seq: RNA-sequencing
RPKM: Reads per kilobase of exon model per million reads
rRNA: Ribosomal RNA
RSEM: RNA-Seq by Expectation Maximization
scRNA-seq: Single-cell RNA-seq
SE read: Single-end read
siRNA: Short-interfering RNA
SNP: Single nucleotide polymorphism
sQTL: Splicing quantitative trait loci
sRNA: Small RNA
TF: Transcription factor
TPM: Transcripts per million
