# Erratum to: A survey of best practices for RNA-seq data analysis

**DOI:** 10.1186/s13059-016-1047-4

**Published:** 2016-08-26

**Authors:** Ana Conesa, Pedro Madrigal, Sonia Tarazona, David Gomez-Cabrero, Alejandra Cervera, Andrew McPherson, Michal Wojciech Szcześniak, Daniel J. Gaffney, Laura L. Elo, Xuegong Zhang, Ali Mortazavi

**Affiliations:** 1 Institute for Food and Agricultural Sciences, Department of Microbiology and Cell Science, University of Florida, Gainesville, FL 32603 USA; 2 Centro de Investigación Príncipe Felipe, Genomics of Gene Expression Laboratory, 46012 Valencia, Spain; 3 Wellcome Trust Sanger Institute, Wellcome Trust Genome Campus, Hinxton, Cambridge, CB10 1SA UK; 4 Wellcome Trust-Medical Research Council Cambridge Stem Cell Institute, Anne McLaren Laboratory for Regenerative Medicine, Department of Surgery, University of Cambridge, Cambridge, CB2 0SZ UK; 5 Department of Applied Statistics, Operations Research and Quality, Universidad Politécnica de Valencia, 46020, Valencia, Spain; 6 Unit of Computational Medicine, Department of Medicine, Karolinska Institutet, Karolinska University Hospital, 171 77 Stockholm, Sweden; 7 Center for Molecular Medicine, Karolinska Institutet, 17177 Stockholm, Sweden; 8 Unit of Clinical Epidemiology, Department of Medicine, Karolinska University Hospital, L8, 17176 Stockholm, Sweden; 9 Science for Life Laboratory, 17121 Solna, Sweden; 10 Systems Biology Laboratory, Institute of Biomedicine and Genome-Scale Biology Research Program, University of Helsinki, 00014 Helsinki, Finland; 11 School of Computing Science, Simon Fraser University, Burnaby, V5A 1S6BC Canada; 12 Department of Bioinformatics, Institute of Molecular Biology and Biotechnology, Adam Mickiewicz University in Poznań, 61-614 Poznań, Poland; 13 Turku Centre for Biotechnology, University of Turku and Åbo Akademi University, FI-20520 Turku, Finland; 14 Key Lab of Bioinformatics/Bioinformatics Division, TNLIST and Department of Automation, Tsinghua University, Beijing, 100084 China; 15 School of Life Sciences, Tsinghua University, Beijing, 100084 China; 16 Department of Developmental and Cell Biology, University of California, Irvine, Irvine, CA 92697-2300 USA; 17 Center for Complex Biological Systems, University of California, Irvine, Irvine, CA 92697 USA

## Erratum

During editing of the article by Conesa *et al.* [1], an error was introduced to some of the citations, such that incorrect references were provided for some articles the second time they were cited. The following sentences are affected:

*Algorithms that quantify expression from transcriptome mappings include RSEM (RNA-Seq by Expectation Maximization) [40], eXpress [41], Sailfish [35] and kallisto [42] among others. These methods allocate multi-mapping reads among transcript and output within-sample normalized values corrected for sequencing biases [35, 41, 43].*

The citation for Sailfish should be [34] (Patro *et al.*, Nat Biotechnol. 2014;32:463–4) in both sentences.

*Additional factors that interfere with intra-sample comparisons include changes in transcript length across samples or conditions [50], positional biases in coverage along the transcript (which are accounted for in Cufflinks), average fragment size [43], and the GC contents of genes (corrected in the EDAseq package [21]).*

The citation for EDAseq should be [20] (Risso *et al.* BMC Bioinformatics. 2011;12:480)

*The NOISeq R package [20] contains a wide variety of diagnostic plots to identify sources of biases in RNA-seq data and to apply appropriate normalization procedures in each case.*

The citation for NOISeq should be [19] (Tarazona *et al.* Nucleic Acids Res. 2015;43:e140)

*These effects can be minimized by appropriate experimental design [51] or, alternatively, removed by batch-correction methods such as COMBAT [52] or ARSyN [20, 53].*

The citations for ARSyN should be [19, 53] (Tarazona *et al.* Nucleic Acids Res. 2015;43:e140, Nueda *et al.* Biostatistics. 2012;13:553–66).

*All these approaches are generally hampered by the intrinsic limitations of short-read sequencing for accurate identification at the isoform level, as discussed in the RNA-seq Genome Annotation Assessment Project paper [30].*

The citation for the RGASP article should be [29] (Engström *et al.* Nat Methods. 2013;10:1185–91).

*We refer the reader to [30] for a comprehensive comparison of RNA-seq mappers.*

This citation should be [29] (Engström et al. Nat Methods. 2013;10:1185–91).
