# Supplementary material for: A survey of best practices for RNA-seq data analysis
Source: Genome Biol. 2016 Jan 26;17:13. doi: 10.1186/s13059-016-0881-8 (PMC4728800; doi:10.1186/s13059-016-0881-8)
Supplement: Additional file 1: Figure S1. — Screenshots of RNA-seq data visualization. a Integrative Genomics Viewer (IGV) [77] display of a gene detected as differentially expressed between the two groups of samples by DEGseq [62]. The bottom track in the right panel is the gene annotation. The tracks are five samples from each group. b RNAseqViewer [80] display of the same data as in (a). c RNAseqViewer heatmap display of a gene detected as differentially spliced between two groups by both DSGSeq [70] and DEXSeq [69]. Introns are hidden in the display to emphasize the signals on the exons. d MISO [81] display of another gene detected as differentially spliced, with junction reads illustrated. (PDF 1152 kb) [file 13059_2016_881_MOESM1_ESM.pdf]

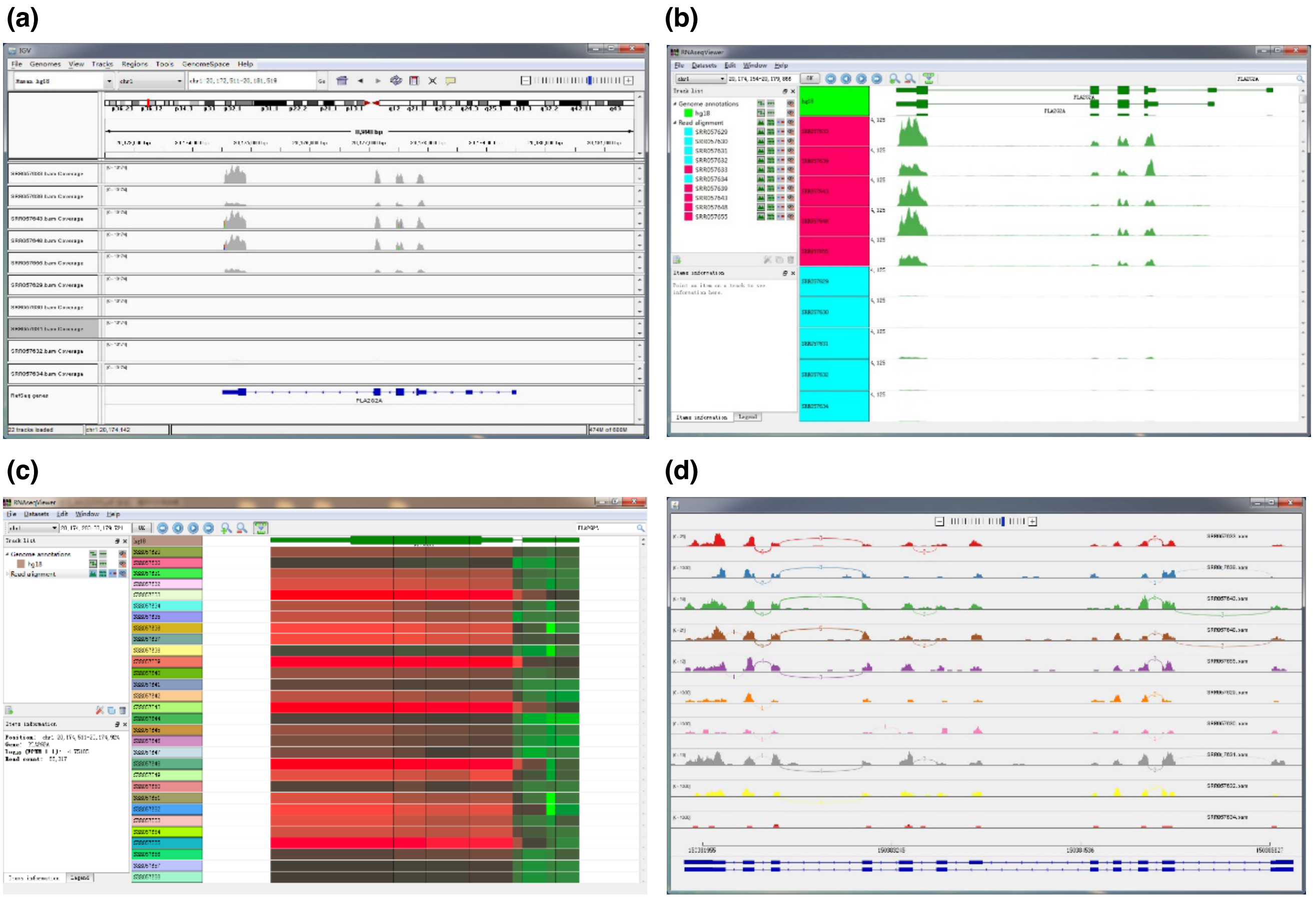
(a)
IGV
PLA2G2A
(b)
RNAseqViewer
(c)
RNAseqViewer
(d)
